# Pennsylvania

**Published:** 1901-12

**Authors:** 


### LEGISLATION.

### PENNSYLVANIA.

### AGRICULTURAL INTERESTS.

Lighter restrictions on the manufacture and sale of oleomargarine, butterine, and similar products are the most important among several laws bearing on agricultural interests. The law prohibits the manufacture and sale of the substance colored in imitation of butter, and specifies license fees to be paid for selling it for what it is in stores and serving it in hotels, restaurants, and boarding-houses. Any citizen, in the State's name, can prosecute for infraction of the law, and will receive half of the fine. He shall also be entitled to recover from the defendant his witness fees and other legal costs.

It will be a misdemeanor to sell, or possess with intent to sell, for human consumption, milk or cream to which has been added boric acid salt, boric acid, salicylic acid, salicylate of soda, formaline, formaldehyde, sodium fluoride, sodium benzoate, or any other substance for the purpose of preserving or coloring.

Provision is made to prevent the spread of disease from carcasses of animals dying of virulent ailments or killed while afflicted with such diseases. The carcasses are to be safely disposed of or destroyed. The State Live-stock Sanitary Board shall enforce this law.

The Agricultural Department is directed to enforce a law against mixing any mineral or substance injurious to the health of domestic animals with feeding stuffs sold or offered for sale. The act defines and regulates the sale of concentrated commercial feeding stuffs. Another act regulates the manufacture and sale of commercial fertilizers.

The importation and sale of dressed carcasses of lambs and sheep with the hoofs on are prohibited. All baled hay and straw must be properly bound and plainly marked with the correct weight.

